# Medical students’ perceptions of international accreditation

**DOI:** 10.5116/ijme.5610.3116

**Published:** 2015-10-11

**Authors:** Halah Ibrahim, Sawsan Abdel-Razig, Satish C Nair

**Affiliations:** 1 Tawam Hospital, Department of Academic Affairs, Abu Dhabi, UAE; 2 Langone School of Medicine, New York University, USA

**Keywords:** accreditation, international medical education, graduate medical education

## Abstract

**Objectives:**

This study aimed to explore the perceptions of medical students in a developing medical education system towards international accreditation.

**Methods:**

Applicants to an Internal Medicine residency program in an academic medical center in the United Arab Emirates (UAE) accredited by the Accreditation Council for Graduate Medical Education-International (ACGME-I) were surveyed between May and June 2014. The authors analysed responses using inductive qualitative thematic analysis to identify emergent themes.

**Results:**

Seventy-eight of 96 applicants (81%) completed the survey. The vast majority of respondents 74 (95%) reported that ACGME-I accreditation was an important factor in selecting a residency program. Five major themes were identified, namely improving the quality of education, increasing opportunities, meeting high international standards, improving program structure, and improving patient care. Seven (10%) of respondents felt they would be in a position to pursue fellowship training or future employment in the United States upon graduation from an ACGME-I program.

**Conclusions:**

UAE trainees have an overwhelmingly positive perception of international accreditation, with an emphasis on improving the quality of training provided. Misperceptions, however, exist about potential opportunities available to graduates of ACGME-I programs. As more countries adopt the standards of the ACGME-I or other international accrediting bodies, it is important to recognize and foster trainee “buy-in” of educational reform initiatives.

## Introduction

As the global medical community becomes increasingly interconnected, there is a growing need for healthcare workers who can provide consistent, high quality patient care.^1^ Worldwide, however, the competencies of graduating physicians can vary considerably from one country to another.^1^ It has been recognized that accreditation systems can be powerful change agents, promoting reform and quality improvement in medical education.^2^ In 2004, the World Health Organization and World Federation for Medical Education strategically partnered to develop standards for accreditation of basic medical education.^3^^,^^4^ Many nations have since instituted uniform accreditation standards as a means of ensuring that minimum quality assurance measures are met. The positive influence of accreditation has been cited in numerous countries.^5^^,^^6^ In 2009, the Taiwan Medical Accreditation Council noted significant improvements in the standards of medical training within a few years of the development of a national accreditation system.^5^ Similarly, it has been documented that students from accredited medical schools in Mexico and the Philippines have demonstrated improved USMLE first time pass rates.^6^ Critics, however, claim that causality has never been proven between accreditation and quality medical education.^7^ Others have argued that the process of accreditation is time-consuming, laborious, and may even detract from education.^8^^,^^9^ 

As the debate continues in the undergraduate arena, postgraduate medical training programs have quickly adopted accreditation standards as a means of standardizing and defining the competencies expected of graduating physicians.^10^ In recent years, Western regulatory bodies, including the United States-based Accreditation Council for Graduate Medical Education- International (ACGME-I), the Royal College of Physicians and Surgeons of Canada and several of the United Kingdom-based Royal Colleges, have expanded their global presence, producing international accreditation standards for nations with developing healthcare and medical education systems. Faced with increasing societal demand for competent locally trained physicians, several countries, including Lebanon, Oman, Qatar, Singapore, and the United Arab Emirates (UAE), have recently adopted the training standards of the Accreditation Council for Graduate Medical Education-International (ACGME-I).^10^^-^^12^

Historically, the decision to pursue accreditation has been instituted in a top-down fashion- mandated by government regulators, and implemented by institutional leaders and teaching faculty,^11^ with little or no input from trainees.  It is becoming increasingly evident, however, that trainees have a different vantage point and contribute valuably to educational reform discussions.^13^ To our knowledge, there are no published data regarding trainee perceptions of international graduate medical education (GME) accreditation. The purpose of this study was to explore the perceptions of medical students towards ACGME-I accreditation.

## Methods

### Setting and participants

The UAE is a small nation in the Middle East bordering the Persian Gulf. Over the past 40 years, the country has experienced economic prosperity with resultant advances in multiple sectors, including healthcare and education. Since 2010, residency training programs in Abu Dhabi, UAE have transitioned to competency-based medical education and sought international accreditation from the ACGME-I.^12^

Convenience sampling methodology was used in this study. Survey respondents included all applicants to a large, well-established Internal Medicine residency program in an academic medical center in Abu Dhabi, UAE. The residency program had received international accreditation by the ACGME-I in 2013. Applicants to the residency program included graduates from medical schools throughout the Arab world and Asia.

### Data collection

A brief survey was developed by the authors and aimed to explore medical student perceptions of potential benefits or implications of international accreditation on their training. The questionnaire was piloted in February 2014 among 20 current residents to ensure comprehension of the questions. These individuals did not participate in the subsequent survey. The survey collected basic demographic information of respondents and included 2 open-ended questions intended to gauge whether accreditation by the ACGME-I played a role in medical students’ selection of residency training programs, as well as students’ perceptions of the potential impact international accreditation on their education. Between May and June 2014, the survey was administered at the end of each residency interview by a volunteer hospital administrator who was not involved in the study or the residency program interview process. Applicants were informed in writing that participation would not affect the residency application or interview process. Survey participation was voluntary and anonymous. The study was approved by the Al Ain District Research Ethics Committee.

### Analysis

Surveys were analysed using inductive qualitative thematic analysis^14^ by 2 members of the research team (HI and SA). SA and HI independently reviewed all surveys collected for patterns and generated codes for each pattern identified. Codes were collated into themes by each investigator. The investigators then convened to review the themes identified. Common themes were selected, named and defined. Discrepancies were reconciled through further review of the surveys and discussion.

## Results

Of the 96 applicants interviewed, 78 completed the survey (81% response rate). Participants were primarily female n=58 (74%) and the vast majority were graduates of medical schools from the UAE and neighbouring Arab countries (Table 1). 

Seventy four of survey participants (95%) reported that ACGME-I accreditation was an important factor in selecting residency training programs. Of these 74 respondents, 71 provided free text responses. Qualitative analysis of survey narratives revealed 5 major themes (Table 2).

Overall, the data show a predominantly positive perspective towards accreditation. Half of the respondents 36 (51%) felt that international accreditation would improve the quality of their education, culminating in the graduation of a highly competent physician. Another prevailing theme was related to perceptions of accreditation affording increased opportunities. Survey respondents felt that research opportunities, as well as future training and career opportunities, would be enhanced for graduates of internationally accredited programs. Seven (10%) of respondents felt that they would be in a better position to pursue fellowship training or future employment in the United States upon graduation from an ACGME-I accredited program. Other themes identified included improving program structure, ensuring high international standards of education, and improving patient care.

**Table 1:** Demographic data of medical student (n= 78)

Applicants to a UAE residency program		
Variable		n (%)
Gender	Male Female	20 (26) 58 (74 )
Age	Mean +/- SD	26.3 +/- 2.83
Marital Status	Single Married	53 (68 ) 25 (32 )
Country of Medical School	UAE ^*^Non-UAE	27 (35 ) 51 (65)

*Countries include Bangladesh, Comoros, Egypt, India, Iraq, Jordan, Pakistan, Palestine, Saudi Arabia, Sri Lanka, Sudan, Syria, and Yemen.

**Table 2:** Themes generated from medical student surveys

Theme	n (%)	Example Respondent Quotes
Quality in education	36 (51)	*“ACGME-I accreditation means that the program will focus on residents’ educational needs”*. Male applicant from Jordan *“I am sure that I will get all the teaching and support that I will need to become a great physician”.* Female applicant from UAE
Increased opportunities	25 (35)	*“The program will allow me more time and opportunities to do research”.* Female applicant from Syria *“After graduating from an ACGME-I accredited program, I will be a better candidate for fellowship positions and job opportunities in America”.* Male applicant from India
Meets high international standards	24 (34)	*“ACGME-I accreditation means that the hospital meets international standards for graduate medical education, focusing on academic excellence and resident well-being”. * Male applicant from Sudan *“ACGME-I accreditation means that the hospital meets international standards for education, research and clinical care”.* Female applicant from UAE
Improved structure	15 (21)	*“ACGME-I accreditation means that the program will be as structured and academically challenging as programs in the USA”.* Female applicant from Yemen
Better patient care	9 (13)	*“We will learn up-to-date and evidence-based practices that will help us provide the best care to our patients”.* Female applicant from Pakistan *“In addition to medical knowledge, we will learn professionalism and communication skills, which will enable us to provide the best care to our patients”.* Female applicant from Egypt

## Discussion

The accreditation of medical education programs has become increasingly popular in both developed and developing countries worldwide.^1^ Given the substantial investment in the training of future physicians and the potential impact of accreditation systems on educational programs, it is important to understand the perceptions of trainees towards accreditation. This is particularly important when considering that educational interventions are associated with improved learning outcomes and success if supported by the trainees.^15^

This is the first study looking at trainee perceptions of international GME accreditation. In our study, the vast majority of respondents considered ACGME-I accreditation an important factor in their selection of a residency program. Study results indicate an overwhelmingly positive perception of accreditation, with an emphasis on improving the quality of training provided. It is concerning, however, that several respondents felt that international accreditation by the ACGME-I would improve their chances of pursuing fellowship or job opportunities in the US. To date, there are no policies facilitating entry of international trainees into the US or other Western physician workforce. International institutions and programs will need to educate their trainees about the meaning of accreditation in order to avoid misperceptions and false hopes. Additionally, these perceptions are paradoxical to the overall objective of many countries who invest in improving accreditation standards as a strategy to retain locally-trained physicians, an essential component of building a competent local healthcare workforce.^10^

It is interesting to note that only a minority of applicants associated accreditation with the penultimate outcome of quality training- improved patient care. This may indicate a more learner-centric view of the value of education.

The primary limitation of this study is related to sample selection. Study participants were restricted to applicants to only one residency training program, potentially limiting the generalizability of findings. These applicants were, however, of diverse nationalities and medical school backgrounds consisting of graduates from countries throughout the Arab world. In fact, this demographic make-up, including the predominance of female respondents, reflects the regional pool of residency applicants, and is likely representative of this population of trainees. Another potential limitation of this study is related to the setting in which the survey was distributed. Since respondents were surveyed during their residency interview process, we attempted to minimize the effect of social desirability response bias by emphasizing the confidentiality and anonymity of data analysis.

## Conclusion

Developing medical education systems worldwide are embracing international accreditation of residency programs as a means of improving the quality of GME and ultimately improving the quality of patient care provided. In our study, applicants to residency training had positive perceptions and expectations of ACGME-I accreditation. As more countries adopt the standards of the ACGME-I or other international accrediting bodies, it is important to recognize and foster trainee “buy-in” of educational reform initiatives. Given the positive perceptions of international accreditation, medical educators and healthcare policy makers can expand training positions in accredited programs in an effort to build a competent, locally trained healthcare workforce that will meet population health needs. Long-term follow up of these trainees can assess potential educational impact of the accreditation process. Understanding the perspectives of current trainees through longitudinal studies and follow-up of graduates from accredited programs are potential areas for future research.

### Conflict of Interest

The authors declare that they have no conflict of interest. 
